# Psychological Effects and Medication Adherence among Korean Patients with Inflammatory Bowel Disease during the Coronavirus Disease 2019 Pandemic: A Single-Center Survey

**DOI:** 10.3390/jcm11113034

**Published:** 2022-05-27

**Authors:** Ji Eun Ryu, Sung-Goo Kang, Sung Hoon Jung, Shin Hee Lee, Sang-Bum Kang

**Affiliations:** 1 Division of Gastroenterology, Department of Internal Medicine, Daejeon St. Mary’s Hospital, College of Medicine, The Catholic University of Korea, Daejeon 34943, Korea; 22101260@cmcnu.or.kr (J.E.R.); shleedjsm@cmcnu.or.kr (S.H.L.); 2 Department of Family Medicine, St. Vincent Hospital, College of Medicine, The Catholic University of Korea, Suwon 16247, Korea; 10001951@cmcnu.or.kr; 3 Division of Gastroenterology, Department of Internal Medicine, Eunpyeong St. Mary’s Hospital, College of Medicine, The Catholic University of Korea, Seoul 03312, Korea; 10002060@cmcnu.or.kr

**Keywords:** COVID-19, inflammatory bowel disease, anxiety, depression, medication adherence

## Abstract

Background and Aim. This study evaluated the impact of coronavirus disease 2019 (COVID-19) on the mental health of inflammatory bowel disease (IBD) patients. We quantified anxiety, depression, and medication adherence among IBD patients through a single-center survey in South Korea during the COVID-19 pandemic. Methods. An electronic survey was made available to patients at the IBD clinic in Daejeon St. Mary’s hospital from July 2021 to September 2021. The validated Hospital Anxiety and Depression Scale (HADS) was used to assess depression and anxiety. The Korean version of the Medication Adherence Rating Scale (KMARS) questionnaire was used to assess medication adherence. Results. In total, 407 patients (56.5%; ulcerative colitis, 43.5%; Crohn’s disease) participated in the survey. Among the respondents, 14.5% showed significant anxiety and 26.3% showed significant depression. Female sex, presence of mental disease, unvaccinated status, and the presence of Crohn’s disease were associated with greater risks of anxiety and depression. Among medications, immunomodulators were associated with a greater risk of anxiety. In terms of KMARS, patients reported favorable medication adherence despite the psychological burden of the pandemic. The KMARS score was 7.3 ± 1.5 (mean ± SD) of 10.0 points. High anxiety and depression were associated with a slight decrease in medication adherence. Conclusions. COVID-19 has increased anxiety and depression among IBD patients, whose medication adherence has nevertheless remained good. Furthermore, anxiety and depression were found to have a negative correlation with adherence. Our results provide insights concerning psychological response and medication adherence among IBD patients in South Korea during the COVID-19 pandemic.

## 1. Introduction

The coronavirus disease 2019 (COVID-19) pandemic has influenced multiple aspects of life, including social interactions. High rates of anxiety and depression have been reported [1,2]. The risks of anxiety and depression are significantly higher in inflammatory bowel disease (IBD) patients than in the general population [3]. Factors such as disease severity, treatment noncompliance, and socioeconomic deprivation are associated with increased anxiety and depression [4]. In addition, IBD-related disability negatively affects quality of life. [5] However, good drug adherence can result in lower disability and higher quality of life [6].

During the pandemic, data concerning COVID-19 cases and vaccination are frequently updated. IBD patients are susceptible to psychological problems and reduced medication adherence. Lack of awareness concerning vaccine necessity or existence is a commonly cited reason for not remaining unvaccinated against COVID-19 [7]. Additionally, the number of IBD patients in South Korea has increased in the prior decade, and psychological issues are important among such patients [8]. Compared with non-IBD patients, IBD patients have a higher level of fear concerning the potential for contracting COVID-19 [9,10]. According to a survey conducted by the Korean Association for the Study of intestinal diseases (KASID) in 2021, more than half of IBD patients in South Korea showed a high level of fear of COVID-19 [11].

We evaluated the impact of COVID-19 on the mental well-being and behavior of South Korean patients with IBD. We quantified anxiety, depression, and medication adherence among IBD patients through a single-center survey. We also investigated predictors of increased anxiety and depression. To our knowledge, this is the first study of mental status and medication adherence among IBD patients in South Korea.

## 2. Materials and Methods

### 2.1. Study Design and Patients 

From July to September 2021, 407 outpatients from the IBD clinic in Daejeon St. Mary’s Hospital participated in the survey. Respondents with an underlying diagnosis of IBD and age > 17 years and treatment period more than 6 months were all included. The application of the evaluation battery was in situ. Patients responded to questions related to epidemiologic features, diseases, COVID-19 screening, vaccination, and mental well-being with the provided electronic device after outpatient treatment. To improve the completeness of the survey, clinical research coordinators were assigned to find out blank questions as to outcomes and incomplete questionnaires were excluded from the study. The validated Hospital Anxiety and Depression Scale (HADS) was used to assess anxiety (HADS-A) and depression (HADS-D). The HADS is a 14-item scale with 7 items each for anxiety and depression subscales [12]. Each item is scored from 0 to 3; a total score of >8 indicates significant anxiety or depression. The Korean version of the Medication Adherence Rating Scale (KMARS) was used to assess medication adherence. The parent Medication Adherence Rating Scale was developed by Thompson et al. in 2000 [13,14]. The KMARS is composed of 10 questions, each of which is scored 0 or 1. The total score ranges from 0 to 10; a higher score indicates greater medication adherence. We regarded a KMARS score of >7 as indicative of good adherence. Additionally, a nine-item self-developed questionnaire was used to evaluate behavioral changes during the COVID-19 pandemic. Clinical data were obtained from electronic medical records at the time of survey completion. Surveys were conducted using an electronic device after the provision of informed consent. Information on the COVID-19 situation in Korea was obtained from the following websites; http://ncov.mohw.go.kr (accessed on 20 December 2021), https://ourworldindata.org (accessed on 20 January 2022), https://www.kdca.go.kr (accessed on 10 January 2022).

### 2.2. Data Analysis

Statistical analysis was conducted using Statistical Analysis Software v. 9.4 (SAS Institute, Cary, NC, USA). Continuous variables are shown as means ± standard deviations (SDs); categorical variables are shown as numbers and proportions (%). Between-group comparisons were carried out by independent sample *t*-tests for continuous variables and the χ^2^ test with or without Fisher’s exact test for categorical variables. Univariate and multivariate logistic regression analyses were performed to identify associations of patient characteristics and KMARS score with depression and anxiety. The results are expressed as odds ratios with 95% confidence intervals. *p*-values < 0.05 were considered indicative of statistical significance.

## 3. Results

### 3.1. Patient Characteristics

In total, 407 patients participated in the survey (Table 1). The mean ± SD age of respondents was 41.8 ± 16.4 years, and 68.3% were men. The mean age at IBD diagnosis was 35.3 ± 15.5 years. More than half of the patients had ulcerative colitis (56.5%), and 43.5% of the patients had Crohn’s disease. The patients were undergoing treatment with mesalamine (78.4%), immunomodulators (46.0%), and biologics (39.6%). Only 2.5% of the patients were undergoing treatment with steroids during the study period. Most patients were in remission (94.6%) and 5.4% were experiencing disease flares. Approximately half of the patients (54.3%) were married and 74.8% were employed at the time of the survey. Only 6.2% of the patients had a pre-existing diagnosis of depression or anxiety. During the study period, 47.2% of the patients had undergone COVID-19 testing; 2.1% reported positive results. Among the 45.7% of patients who had been vaccinated against COVID-19, only 46.0% were fully vaccinated.

### 3.2. Mental Well-Being 

The HADS score among the patients was 9.5 ± 6.7 (mean ± SD) (Table 1). Anxiety and depression characteristics are summarized in Table 2. Female sex, unmarried status, unemployed status, presence of mental disease, presence of Crohn’s disease, high disease activity, and unvaccinated status were associated with a high HADS score. COVID-19 testing did not strongly influence the level of anxiety or depression. Female sex, presence of mental disease, and unvaccinated status were associated with significant increases in anxiety and depression. Univariate and multivariate analyses were performed to assess predictors of depression and anxiety (Table 3). Among the respondents, 14.5% showed significant anxiety and 26.3% showed significant depression; 11.8% showed both significant anxiety and depression. Multivariate analysis indicated that female sex, presence of mental disease, unvaccinated status, and presence of Crohn’s disease were associated with greater risks of anxiety and depression. Among oral medications, immunomodulators were associated with a greater risk of anxiety and steroids were associated with a lower risk of depression. Among ulcerative colitis patients, the medications used were (in decreasing order): mesalamine (92.2%), immunomodulators (22.6%), biologics (20.0%), and steroids (2.2%). Among Crohn’s patients, the medications used were immunomodulators (72.9%), biologics (60.5%), mesalamine (57.1%), and steroids (1.7%).

### 3.3. Medication Adherence 

The mean ± SD medication adherence score was 7.3 ± 1.5. Approximately 72.5% of the respondents had a KMARS score of >7 points. In a univariate analysis of the association between the KMARS score and variables, for each 1-point increase in HADS-A score, the KMARS score decreased by 0.102 points; this relationship was statistically significant. With respect to HADS-D, the KMARS score decreased by 0.078 points for each 1-point increase in HADS-D; this relationship was statistically significant. Therefore, greater anxiety or depression were associated with a slight decrease in medication adherence. In addition, for each 1-year increase in the age at diagnosis of IBD, the KMARS score increased by 0.010 points; this relationship was statistically significant. In the multivariate analysis, for each 1-point increase in HADS-A score, the KMARS score decreased by 0.074 points; this relationship was statistically significant. Furthermore, each 1-point increase in HADS-D score resulted in a 0.021-point decrease in KMARS score; however, this relationship was not statistically significant. The KMARS score decreased by 0.352 points in employed patients (Table 4).

### 3.4. Behavioral Changes

The patients reported that the COVID-19 pandemic reduced their time with friends (76.8%), increased their communication with others concerning health (65.5%), caused them to delay tasks (44.6%) and shop for certain types of food (42.9%), reduced their time with family (37.5%), and increased their efforts to access health care (30.4%) and to obtain medication (18.5%) (Table 5). Fewer than 10% of the patients reported increased smoking (8.9%) and alcohol consumption (7.1%).

## 4. Discussion

According to National Health Insurance Service statistics, the number of IBD patients in South Korea increased from 2010 to 2019. Indeed, KASID reported that the number of IBD patients has increased more than twofold during the past decade, thereby necessitating investigations into changes in disease burden and behavioral patterns [8,15]. The psychological burden of the COVID-19 pandemic has been increased by frequent changes in policies, the introduction of vaccination, and reports of adverse events. The negative effect of disease on mental health in IBD patients has been reported. IBD patients are at least threefold and twofold more likely to develop anxiety and depressive disorders, respectively; treatment of such psychological problems can improve the long-term outcomes [16,17].

As of 7 July 2021, 185.55 million cases of COVID-19 had been reported worldwide, with over 4 million confirmed deaths. In South Korea, these figures were 164,028 cases and 2034 deaths [18]. The South Korean government introduced social distancing rules to slow the spread of COVID-19 in March 2020 and implemented a COVID-19 vaccination program on 26 February 2021 [19,20].

At the beginning of this study, the COVID-19 vaccination rate (at least one dose) in South Korea was higher (30.1%) than the global rate (24.9%). However, other countries have higher vaccination rates, including the United Arab Emirates (74.0%), Canada (68.7%), the United Kingdom (66.9%), and the United States (55.8%). In terms of full vaccination, the United Arab Emirates ranks first (64.1%), followed by the United Kingdom (50.1%), and the United States (48.8%); in South Korea, the rate of full vaccination is 11.1%. During the study period, the South Korean government frequently changed the social distancing rules according to the number of domestic confirmed cases, which was updated daily. At the time the survey began, the highest level (level 4) of social distancing rules was in force. Private gatherings of up to three people were prohibited after 18:00 and restaurants were permitted to offer only take-out and delivery after 22:00. Comprehensive social distancing rules affected all areas of daily life including work, education, and sports activities [18,21].

Data concerning the incidences of anxiety and depression during the COVID-19 pandemic among IBD patients in South Korea are scarce. The patients in this study had higher rates of anxiety (14.5%) and depression (26.3%) than patients in a pre-COVID-19 nationwide study (anxiety, 12.2%; depression, 8.0%) [3]. According to previous studies conducted in Portugal and Italy during the COVID-19 pandemic, more than 50% of respondents showed moderate or severe levels of anxiety. [2,22] Our results reported a lower percentage of anxiety and depression. However, unlike previous studies, this study was conducted one year after the outbreak of the COVID-19 pandemic. 

In this study, 6.2% of patients had a previous diagnosis of mental disease. Most of the patients with significant anxiety or depression did not have such a prior diagnosis. The development of such diagnoses could be related to the COVID-19 pandemic, although some patients may have had undiagnosed mental disease before the pandemic.

Multivariate analysis of predictors of significant anxiety or depression can provide insights concerning interventions needed to reduce the mental health burden during the COVID-19 pandemic. Female sex, the presence of mental disease, and the presence of Crohn’s disease were associated with greater risks of anxiety and depression. Our results are different from those of Trindade et al., which showed no differences between Crohn’s disease and ulcerative colitis patients. [22] With respect to COVID-19 vaccination, unvaccinated patients had greater risks of anxiety and depression, presumably because IBD patients were more likely to take any recommended precautions. A meta-analysis reported a lower incidence of COVID-19 infection among IBD patients than among the general population [23]. Uncertainty concerning the benefits and risks of vaccination may make IBD patients more prone to anxiety and depression.

Immunomodulators were associated with a greater risk of anxiety. In South Korea, some IBD patients regard immunomodulators as suppressants. This could cause fear, despite the unclear relationship between immunomodulators and anxiety. Although previous findings reported reduced medication adherence in IBD patients using steroids, our results for steroids cannot inform conclusions because a small proportion of IBD patients used steroids. [24] Therefore, the mental well-being of patients should be considered when making recommendations concerning the cessation of immunomodulators or biologics among IBD patients during the pandemic.

Medication adherence can considerably affect the quality of life and disease control in IBD patients. A four-item version of the Medicine Adherence Report Scale was previously used to assess medication adherence in IBD patients [25,26]. The Medicine Adherence Report Scale total score ranges from 5 to 20, with each statement scored on a 5-point Likert scale, ranging from always [1] to never [5]. Scores of 17 to 20 are considered good adherence [27]. However, we used the KMARS to assess adherence, which comprises more than four questions. Furthermore, respondents answer each question yes or no. However, a reliable KMARS cut-off score for good adherence has not yet been established. Based on a systematic review before the pandemic, the nonadherence rate among IBD patients is 7% to 72%; most studies reported 30% to 45% [28]. Although it is difficult to compare adherence rates before and after the COVID-19 pandemic, our findings indicate that IBD patients showed good medication adherence despite the psychological effects of the COVID-19 pandemic. Several studies in Asia reported medication nonadherence rates among IBD patients of 20% to 30%, similar to our results [29]. Results of the present study also correspond with those of earlier European studies, which reported COVID-19 prevalence did not affect medication adherence in IBD patients [22,30].

The above findings may be explained as follows. First, the KASID educational materials and campaigns could have promoted medication adherence among South Korean IBD patients. The KASID distributed vaccination guidelines for South Korean IBD patients according to their medications [31,32]. Second, the “Band” social media platform (run by the medical team at our IBD Center) provided patients with comprehensive information. This 24 h online platform enabled communication and question–answer interactions between physicians and patients. Because open communication with healthcare providers is important to maintain control of psychological stress, such social media interventions can reduce the psychological burden of patients and improve their medication adherence [33]. 

This study had several limitations. First, it was a single-center, cross-sectional study. However, we enrolled 407 outpatients, a considerable number from a single center. A larger multicenter study involving a larger number of outpatients and hospitalized patients is needed to verify the results. Second, we cannot assume that COVID-19 directly affected the mental well-being of IBD patients. Several factors may have affected the results because this was a self-reported survey study. Third, the possibility of unavoidable bias may exist due to uncontrolled factors. The percentage of having previous mental illness was too low and comparisons were not made with this variable. In addition, the survey was conducted for 14 weeks and the frequent changes in restrictions on public activity promulgated by the South Korean government during that period may have affected the responses.

Nonetheless, this study had several strengths. First, it was a timely study. During the study period, COVID-19 vaccination was in the pipeline in South Korea. Our results provide insights concerning how IBD patients reacted to COVID-19 and the effect (if any) of vaccination. Second, this was the first South Korean study of both mental well-being and medication adherence. Third, we evaluated behavioral changes using a self-developed, albeit unvalidated, questionnaire. The high rates of anxiety and depression in IBD patients indicate the need for the development of effective interventions.

## 5. Conclusions

The COVID-19 pandemic has increased anxiety and depression among IBD patients, whose medication adherence has nevertheless remained good. Furthermore, anxiety and depression were negatively associated with medication adherence. Our results provide insights concerning psychological response and medication adherence among IBD patients in South Korea during the COVID-19 pandemic. 

## Figures and Tables

**Table 1:** Characteristics of patients with IBD responding to the questionnaire.

Characteristic	All (N = 407)
Age (mean ± SD)	41.82 ± 16.35
Gender, *n* (%)	
Male	278 (68.30)
Female	129 (31.70)
Subtype of IBD, *n* (%)	
UC	230 (56.51)
CD	177 (43.49)
PO medication, *n* (%)	
Mesalamine	319 (78.38)
Steroid	10 (2.46)
Immunomodulator	187 (45.95)
Biologics	161 (39.56)
Disease status, *n* (%)	
Remission	385 (94.59)
Flare	22 (5.41)
Age at IBD diagnosis, mean ± SD	35.25 ± 15.54
Marriage status, *n* (%)	
Married	221 (54.30)
Unmarried	186 (45.70)
Job status, *n* (%)	
Employed	303 (74.81)
Unemployed	102 (25.19)
Presence of mental disease, *n* (%)	
Yes	25 (6.16)
No	381 (93.84)
COVID-19 screening test, *n* (%)	
Yes	192 (47.17)
No	215 (52.83)
COVID-19 screening result, *n* (%)	
Positive	5 (2.05)
Negative	239 (97.95)
Vaccination, *n* (%)	
Yes	189 (45.68)
No	220 (54.32)
Vaccination dose, *n* (%)	
1st	102 (53.97)
2nd	87 (46.03)
HADS total score, mean ± SD	9.52 ± 6.70
KMARS total score, mean ± SD	7.28 ± 1.49
Smoking, *n* (%)	
Ex-smoker	109 (29.70)
Never	206 (56.13)
Current smoker	52 (14.17)
Drinking, *n* (%)	
Never	148 (40.22)
Regular drinking	35 (9.51)
Occasional drinking	185 (50.27)

SD, standard deviation; IBD, inflammatory bowel disease; UC, ulcerative colitis; CD, Crohn’s disease; PO, per oral; HADS, hospital anxiety and depression scale; KMARS, Korean version of the medication adherence rating scale.

**Table 2:** Average scores of HADS and KMARS among respondents according to variables.

Variables	HADS Score	*p*-Value	CohenD	HADS Anxiety	*p*-Value	CohenD	HADS Depression	*p*-Value	CohenD	KMARS Score	*p*-Value	CohenD
Gender		0.01	−0.29		<0.001	−0.41		0.18	−0.54		0.73	0.04
Male	8.90 ± 6.50			3.72 ± 3.24			5.18 ± 3.74			7.29 ± 1.54		
Female	10.84 ±6.97			5.12 ± 3.76			5.73 ± 3.83			7.24 ± 1.39		
Marital status		0.07	−0.18		0.16	−0.14		0.06	−0.19		0.40	0.08
Married	8.97 ± 6.57			3.94 ± 3.50			5.04 ± 3.57			7.33 ± 1.56		
Unmarried	10.16 ±6.82			4.42 ± 3.42			5.74 ± 3.97			7.21 ± 1.41		
Job status		0.25	−0.15		0.70	−0.04		0.09	−0.22		0.06	−0.22
Employed	9.25 ± 6.30			4.12 ± 3.38			5.13 ± 3.50			7.21 ± 1.50		
Unemployed	10.24 ±7.79			4.27 ± 3.77			5.96 ± 4.44			7.53 ± 1.39		
Presence of mental disease		<0.001	1.49		<0.001	1.35		<0.001	1.37		0.14	−0.35
Yes	18.32 ±8.59			8.36 ± 5.21			9.96 ± 4.06			6.80 ± 1.68		
No	8.93 ± 6.15			3.88 ± 3.15			5.04 ± 3.55			7.32 ± 1.46		
IBD subtype		0.06	−0.19		0.22	−0.12		0.03	−0.22		0.05	0.20
Ulcerative colitis	8.97 ± 6.43			3.97 ± 3.42			5.00 ± 3.59			7.40 ± 1.42		
Crohn’s disease	10.23 ±6.99			4.40 ± 3.53			5.82 ± 3.96			7.11 ± 1.57		
Disease activity		0.18	−0.29		0.31	−0.30		0.27	−0.24		0.55	0.13
Remission	9.41 ± 6.60			4.10 ± 3.40			5.31 ± 3.74			7.29 ± 1.49		
Flare	11.36 ±8.32			5.14 ± 4.60			6.23 ± 4.28			7.09 ± 1.63		
COVID−19 screening test		0.33	0.10		0.19	0.13		0.61	0.05		0.41	−0.08
Yes	9.86 ± 6.81			4.40 ± 3.63			5.46 ± 3.66			7.21 ± 1.44		
No	9.21 ± 6.61			3.94 ± 3.32			5.27 ± 3.88			7.33 ± 1.54		
Vaccination		0.01	−0.28		0.01	−0.26		0.01	−0.25		0.07	0.18
Yes	8.48 ± 6.17			3.64 ± 3.36			4.83 ± 3.39			7.43 ± 1.47		
No	10.32 ±7.01			4.55 ± 3.49			5.77 ± 4.03			7.16 ± 1.49		

All values are mean ± SD. *p*-values were calculated by the *t*-test. CohenD is Cohen’s D.

**Table 3:** Predictors for moderate depression and anxiety.

	Anxiety				Depression			
Characteristics	Univariate Analysis OR (95% CI)	*p*-Value	Multivariate Analysis OR (95% CI)	*p*-Value	Univariate Analysis OR (95% CI)	*p*-Value	Multivariate Analysis OR (95% CI)	*p*-Value
Age	1 (0.98, 1.02)	0.928	1.03 (0.98, 1.08)	0.318	1 (0.98, 1.01)	0.694	1 (0.96, 1.05)	0.936
Gender (male vs. female)	0.42 (0.24, 0.73)	0.002	0.38 (0.2, 0.71)	0.003	0.67 (0.42, 1.06)	0.087	0.57 (0.34, 0.95)	0.032
Marital status (married vs. unmarried)	1.08 (0.62, 1.88)	0.786	0.97 (0.43, 2.19)	0.941	0.77 (0.5, 1.2)	0.250	0.65 (0.34, 1.24)	0.188
Job status (employed vs. unemployed)	0.81 (0.44, 1.49)	0.488	1.12 (0.55, 2.26)	0.760	0.84 (0.51, 1.39)	0.505	1.16 (0.65, 2.05)	0.616
Presence of mental disease (yes vs. no)	7.89 (3.4, 18.33)	<0.0001	9.39 (3.74, 23.57)	<0.0001	10.69 (4.14, 27.61)	<0.0001	12.77 (4.67, 34.95)	<0.0001
Age at IBD diagnosis	0.99 (0.98, 1.01)	0.523	0.98 (0.94, 1.03)	0.471	1 (0.98, 1.01)	0.809	1.02 (0.97, 1.06)	0.461
COVID-19 screening test (yes vs. no)	1.01 (0.58, 1.76)	0.962	0.94 (0.51, 1.73)	0.844	1.03 (0.66, 1.6)	0.906	0.95 (0.58, 1.54)	0.823
Vaccination (yes vs. no)	0.63 (0.36, 1.13)	0.120	0.4 (0.19, 0.84)	0.016	0.68 (0.43, 1.07)	0.093	0.5 (0.28, 0.89)	0.019
Subtype of IBD		0.344		0.050		0.055		0.034
Ulcerative colitis	0.77 (0.44, 1.33)		0.44 (0.2, 1)		0.65 (0.42, 1.01)		0.5 (0.27, 0.95)	
Crohn’s disease	Index		Index		Index	0.055	Index	
PO medication								
Mesalamine (yes vs. no)	1.15 (0.6, 2.21)	0.671	1.12 (0.47, 2.64)	0.800	1.07 (0.63, 1.81)	0.813	0.7 (0.36, 1.37)	0.293
Steroid (yes vs. no)	1.54 (0.19, 12.38)	0.685	2.88 (0.28, 29.31)	0.371	0.23 (0.06, 0.82)	0.024	0.23 (0.06, 0.92)	0.038
Immunomodulator (yes vs. no)	1.4 (0.79, 2.45)	0.247	2.35 (1.08, 5.12)	0.031	1.01 (0.65, 1.57)	0.971	1.49 (0.81, 2.74)	0.205
Biologics (yes vs. no)	1.59 (0.88, 2.88)	0.127	1.62 (0.77, 3.4)	0.206	0.97 (0.62, 1.51)	0.877	0.86 (0.49, 1.51)	0.595

OR, odds ratio; CI, confidence interval.

**Table 4:** Association between variables and Korean version of Medication Adherence Rating Scale (KMARS).

	Univariate Analysis			Multivariate Analysis		
	β	95% CI	*p*-Value	β	95% CI	*p*-Value
HADS Anxiety	−0.102	−0.142 to −0.061	<0.0001	−0.074	−0.135 to −0.013	0.018
HADS Depression	−0.078	−0.116 to −0.040	<0.0001	−0.021	−0.076 to 0.035	0.466
Gender (male vs. female)	0.055	−0.258 to 0.368	0.732	0.006	−0.312 to 0.324	0.972
Marital status (married vs. unmarried)	0.125	−0.167 to 0.417	0.400	−0.045	−0.403 to 0.313	0.804
Job status (employed vs. unemployed)	−0.318	−0.650 to 0.014	0.060	−0.352	−0.695 to −0.010	0.044
Presence of mental disease (yes vs. no)	−0.520	−1.120 to 0.078	0.089	−0.069	−0.702 to 0.564	0.830
Age at IBD diagnosis	0.010	0.001 to 0.020	0.031	0.003	−0.010 to 0.016	0.678
COVID−19 screening test (yes vs. no)	−0.121	−0.413 to 0.170	0.414	−0.043	−0.330 to 0.244	0.768
Vaccination (yes vs. no)	0.269	−0.022 to 0.560	0.070	0.137	−0.188 to 0.461	0.409
Subtype of IBD (Ulcerative colitis vs. Crohn’s disease)	0.291	−0.001 to 0.584	0.051	0.181	−0.199 to 0.561	0.350
PO medication						
Mesalamine (yes vs. no)	0.238	−0.115 to 0.591	0.186	0.071	−0.328 to 0.471	0.726
Steroid (yes vs. no)	0.023	−0.918 to 0.964	0.962	0.032	−0.889 to 0.952	0.946
Immunomodulator (yes vs. no)	−0.256	−0.548 to 0.035	0.084	−0.153	−0.508 to 0.202	0.398
Biologics (yes vs. no)	0.034	−0.264 to 0.332	0.823	0.203	−0.134 to 0.540	0.236

**Table 5:** Impact of COVID-19 on behaviors among patients with inflammatory bowel disease.

Variables	*n* * (%)
Reduced time with friends	129 (76.8)
More communication with people about health	110 (65.5)
Delay tasks to do	75 (44.6)
Shopping for certain types of food	72 (42.9)
Reduced time with family	63 (37.5)
Increased efforts to access health care service	51 (30.4)
Increased efforts to obtain medication	31 (18.5)
Increased frequency to smoke	15 (8.9)
Increased frequency to drink alcohol	12 (7.1)

* Participants who reported “Yes” to each question.

## Data Availability

Data can be made available from the corresponding author upon request.
